# Meta-analysis of the correlation between high expression of lncRNA NEAT1 in rectal cancer and pathological features and prognosis

**DOI:** 10.5937/jomb0-47889

**Published:** 2024-06-15

**Authors:** Qiyi Lin, Jianpeng Pan, Huaishuai Wang, Yinlin Li, Yixiang Zhuang, Zhicong Cai, Gaofeng Lin, Weibo Liu, Guoxi Xu

**Affiliations:** 1 Jinjiang Municipal Hospital, Department of Gastrointestinal Surgery, Quanzhou, China

**Keywords:** lncRNA NEAT1, prognosis, rectal cancer, meta analysis, lncRNA NEAT1, prognoza, rak rektuma, meta analiza

## Abstract

**Background:**

To systematically evaluate the relationship between the expression level of long noncoding RNA NEAT1 and the clinical characteristics and prognostic value of rectal cancer patients.

**Methods:**

PubMed, EMBASE, Cochrane library database and case-control studies on the correlation between abnormal expression of lncRNA NEAT1 and prognosis of rectal cancer patients published by the American clinical trials registry before May 1, 2023 were searched. The search time was from the establishment of the database to May 30, 2023.

**Results:**

A total of 7 case-control studies were included, including 1063 cancer patients. The results of meta-analysis showed that the high expression of lncRNA NEAT1 was significantly correlated with the degree of differentiation [or=0.45, 95%CI=0.32-0.63, P<0.01], tumor size [or=0.59, 95%CI=0.42-0.82, P<0.01], and overall survival [HR=1.34, 95%CI=1.21-1.48, P<0.001]; However, it was not associated with gender [or=1.23, 95%CI= 0.88-1.72, P=0.23] and lymph node metastasis [or=0.87, 95%CI=0.45-1.66, P=0.67].

**Conclusions:**

The high expression of lncRNA NEAT1 may be a risk factor for poor prognosis in patients with malignant tumors, and lncRNA NEAT1 can be used as a potential biomarker to evaluate its prognosis.

## Abstract (sr)

**Uvod:**

Cilj je bio da se sistematski proceni odnos između nivoa ekspresije duge nekodirajuće RNK NEAT1 i kliničkih karakteristika i prognostičke vrednosti pacijenata sa rakom rektuma.

**Metode:**

Pretražene su PubMed, EMBASE, Cochrane bibliotečka baza podataka i studije slučaja-kontrole o korelaciji između abnormalne ekspresije lncRNA NEAT1 i prognoze pacijenata sa rakom rektuma koje je objavio američki registar kliničkih ispitivanja pre 1. maja 2023. godine. Vreme pretrage je bilo od uspostavljanja baze podataka do 30. maja 2023. godine.

**Rezultati:**

Uključeno je ukupno 7 studija slučaj-kontrola, uključujući 1063 pacijenata sa rakom. Rezultati meta-analize su pokazali da je visoka ekspresija lncRNA NEAT1 u značajnoj korelaciji sa stepenom diferencijacije [or=0,45, 95%CI=0,32-0,63, P<0,01], veličinom tumora [ili=0,59, 95%CI=0,42-0,82, P<0,01] i ukupno preživljavanje [HR=1,34, 95% CI=1,21-1,48, P<0,001]; Međutim, nije bilo povezano sa polom [ili=1,23, 95%CI=0,88-1,72, P=0,23] i metastazama u limfnim čvorovima [ili=0,87, 95% CI =0,45-1,66, P=0,67].

**Zaključak:**

Visoka ekspresija lncRNA NEAT1 može biti faktor rizika za lošu prognozu kod pacijenata sa malignim tumorima, a lncRNA NEAT1 se može koristiti kao potencijalni biomarker za procenu njegove prognoze.

## Introduction

Rectal cancer is one of the most common malignancies affecting the digestive system and is the third leading cause of cancer-related deaths. Currently, the main treatment options for rectal cancer include surgical resection, chemotherapy and radiotherapy [1]. In China, where the incidence of colorectal cancer has also been gradually increasing in recent years, new treatments for primary and metastatic colorectal cancer have emerged, offering patients more options; these include laparoscopic surgery for primary disease, more aggressive resection of metastatic disease (e.g. liver and lung metastases), radiotherapy for rectal cancer, and neoadjuvant and palliative chemotherapy [2]. However, although the above treatment options are more effective measures in the clinical management of rectal cancer, prolonging survival and leading to some improvement in the quality of survival, they still do not improve the 5-year survival rate of patients.

Despite advances in rectal cancer screening tests and treatment strategies, clinical outcomes for rectal cancer remain poor. Local recurrence or distant metastases are the main cause of poor prognosis in colorectal cancer. Although the tumour, lymph node, metastasis (TNM) staging system provides important and useful guidelines for selecting treatment, patients at a similar TNM stage may have a completely different clinical outcome. There is therefore an urgent need to develop novel biomarkers for the diagnosis and prognosis of colorectal cancer [3].

Long noncoding RNAs (lncRNAs) are a class of RNA molecules longer than 200 nucleotides that do not encode proteins. They play a crucial role in cancer biology, acting as key regulators in various cellular processes such as cell growth, differentiation, and apoptosis. LncRNAs are involved in gene expression regulation at the epigenetic, transcriptional, and posttranscriptional levels. Their dysregulation has been linked to the development and progression of various cancers, including colorectal cancer [4]. With the development and advancement of RNA sequencing technology and transcriptome analysis, numerous studies [5] have found that lncRNAs are also involved in the abnormal regulation of cancer. Nuclear paraspeckle assembly transcript1 (NEAT1), a novel lncRNA identified by Hutchinson et al. [6] in 2007, consists of 3729 nucleotides and is located on the chromosome 11 locus in tumour syndrome multiple endocrine adenomas, and its association with PSP1, p54nrb and SFPQ, which together maintain the morphology and function of paranuclear spots. lncRNA NEAT1 is mainly expressed in ovarian, prostate, colon and pancreatic tissues [7] and is mainly involved in physiological processes such as corpus luteum and mammary gland development, epigenetic regulation, stress response and immune response [8]. In recent years, numerous studies [9] have found that NEAT1 is dysregulated in expression in a variety of solid tumours and is involved in tumour cell proliferation, apoptosis, invasion, metastasis and chemoresistance, and is associated with poor prognosis in tumour patients. Clearly, NEAT1 may be an important potential biomarker and clinical therapeutic target for cancer. However, the relationship between NEAT1 expression levels and prognosis and clinicopathological features in gastrointestinal malignancies remains unclear, and some studies have shown conflicting results. For example, Li et al. [10] found that high NEAT1 expression was a poor prognostic factor in colorectal cancer, and the relationship between NEAT1 expression and prognosis in colorectal cancer was analysed by multivariate analysis, but the results suggested no statistical significance. Similarly, there is controversy regarding the correlation between clinicopathological features such as lymph node metastasis, degree of tumour differentiation and NEAT1 expression.

This investigation is crucial for enhancing our understanding of NEAT1’s role in rectal cancer, which could have significant diagnostic and therapeutic implications. The findings may establish NEAT1 as a potential biomarker for early detection and patient prognosis, guiding more personalized treatment strategies. Additionally, if NEAT1 is implicated in disease progression or treatment resistance, it could be targeted in new therapeutic approaches. This study not only contributes to the current knowledge of rectal cancer but also sets a foundation for future research into the role of lncRNAs in oncology, potentially leading to innovative treatments and improved patient outcomes in rectal cancer and beyond.

Our study aims to resolve these discrepancies by conducting a comprehensive meta-analysis of existing research on NEAT1 in colorectal cancer. This approach is novel in its application and significant in its potential to clarify the prognostic value of NEAT1 expression in colorectal cancer. By systematically analyzing previous studies, we seek to establish a more definitive correlation between NEAT1 expression levels and the progression of colorectal cancer. The findings of this study could not only provide a deeper understanding of the molecular mechanisms underlying colorectal cancer but also pave the way for the development of innovative diagnostic tools and more effective therapeutic strategies, thereby enhancing patient outcomes and survival rates.

## Materials and methods

### Search strategy

The literature search was conducted in May 2023 in Pubmed, Embase, Cochrane library databases and the US Clinical Trials Registry with indexing terms related to enteral nutrition and prone position. The main MesH used in the search were as follows: “lncRNA, Cancer, Tumor, Neoplasm, Rectal, NEAT1, Nuclear Paraspeckle Assembly transcript 1”, with no restrictions on language or date of publication. There were no restrictions on the language or date of publication and the search was updated in May 2023.

### Literature inclusion and exclusion criteria

Inclusion criteria: (1) Study type: cohort study; (2) Study population: studies with a clear pathological diagnosis of gastrointestinal malignancy, no language restriction was imposed on the included literature; (3) Studies exploring the relationship between lncRNA NEAT1 and the prognosis or clinicopathological features of gastrointestinal malignancy; (4) Risk ratio (HR) and its 95% confidence interval (CI) were given in the text. Confidence interval (CI) are given in the text or can be obtained by sufficient information calculation; (5) The expression level of lncRNA NEAT1 distinguishes between high and low expression.

Exclusion criteria: 1. Studies of clinical randomised controlled trials of enteral and parenteral nutrition therapy in non-critically ventilated patients; 2. Duplicate publications, conference papers, poor quality literature; 3. Full text still not available by all means; 4. Animal experiments; 5. Those who did not meet the criteria.

### Literature screening and data extraction

All relevant literature initially retrieved was collated by extracting information according to the above inclusion and exclusion criteria. Researchers who had received training in evidence-based methods independently screened literature and extracted information according to the inclusion and exclusion criteria, and cross-checked, and if differences of opinion arose, they reached agreement through discussion or assisted with another researcher to adjudicate. This included the inclusion test method, sample size, NEAT1 high expression rate, intercept values, outcome indicators, follow-up time, and clinicopathological characteristics (including age, gender, degree of tumour differentiation, tumour diameter, clinical stage, lymph node metastasis, etc.). All literature eligible for the study was repeatedly screened according to the above criteria, and those who did not meet the criteria after secondary screening were excluded, and the final study literature that met the inclusion criteria was finally counted.

### Literature quality evaluation

The risk of bias assessment tool recommended by Cochrane evaluation manual 5.1.0 was used to evaluate the quality of randomized controlled trials [11]. This tool scrutinizes several key areas: random sequence generation and allocation concealment to prevent selection bias; blinding of participants, personnel, and outcome assessors to avoid performance and detection biases; assessment of incomplete outcome data to ensure internal validity; and examination of selective reporting to avoid reporting bias. Each study was rated across these domains as having low, high, or unclear risk of bias. This rigorous assessment process enhances the reliability and validity of the meta-analysis by ensuring that only high-quality studies contribute to the overall findings, thus strengthening the credibility and robustness of the conclusions drawn from the analyzed trials.

### Statistical analysis

Revman 5.2 software was used for meta-analysis. The heterogeneity of the included studies was evaluated by the I^2^ statistic and chi^2^. In the meta-analysis, heterogeneity among studies was assessed using the Chi-squared test (Chi^2^) and the I^2^ statistic. A Chi^2^ p-value of less than 0.10 was set as the threshold for significant heterogeneity, acknowledging the test’s low power with smaller numbers of studies. The I^2^ statistic was used to quantify heterogeneity, with values interpreted as follows: 0–40% indicating low, 30–60% moderate, 50–90% substantial, and 75–100% considerable heterogeneity. Significant heterogeneity was identified at an I2 value greater than 50%, prompting the use of random-effects models. At this time, the random effect model was used for combined analysis, otherwise the fixed effect model was used. The odds ratio (RR) and its 95% confidence interval (CI) were used as the effect indicators of the study. In order to ensure the stability of the results, sensitivity analysis was performed by excluding the reanalysis of individual studies. In addition, the degree of publication bias was evaluated by observing the funnel plot.

## Results

### Literature search results

**Figure 1:**
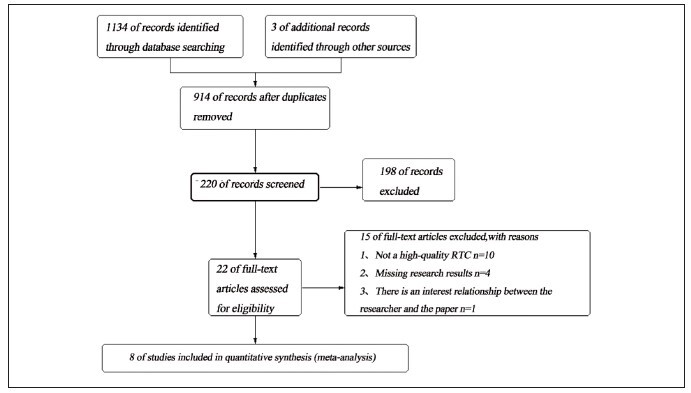
Flow chart of literature screening.

**Figure 2:**
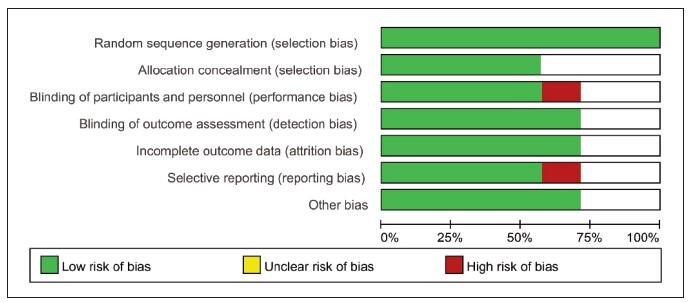
Summary chart of risk bias.

The preliminary search obtained 1137 articles, and 3 related articles were supplemented through other ways, a total of 1137 articles. 914 articles were obtained by endnote X9 software after excluding duplicate articles. After reading the title, abstract and full text, the articles that did not meet the inclusion criteria were excluded, and 7 articles were finally included. The screening process is shown in Figure 1. The risk of bias of the included studies was assessed through the evaluation table recommended by the Co-chrane reviewer manual. All the included studies were of high quality, and the implementation bias, measurement bias and follow-up bias all had low risk of bias (Figure 2).

### Basic characteristics of literature

The specific basic characteristics of the included literatures are shown in Table 1. Finally, a total of 7 literatures ([10] [12] [13] [14] [15] [16] [17]) including 1063 patients were included in the meta-analysis. The publication time of the included literatures was from 2015 to 2021. The study population was all China. The sample size of each study varied from 56 cases to 239 cases. The detection method was QRT PCR. The intercept values of NEAT1 expression high and low groups included ROC curve, median value, and average value; Although some studies did not give a clear cut-off value of NEAT1 expression, they distinguished high and low expression groups. A total of 7 studies evaluated the relationship between NEAT1 and overall survival and provided relevant information on clinicopathological characteristics.

**Table 1:** Basic characteristics of the included literature.

Author (year)	Country	Specimen	Detection<br>method	Sample<br>(cases)	High<br>expression ratio	Intercept value	Outcome<br>measures	HR(95% CI)
Li (2015) [10]	China	Tissue	qRT-PCR	239	46.03%	T/N, 2	OS, DFS	1.70 (1.18~2.45)
Peng (2017) [12]	China	Tissue	qRT-PCR	56	55.36%	NA	OS, DFS	1.45 (1.18~4.61)
Zhang (2018) [13]	China	Tissue	qRT-PCR	71	50.7%	Average value	OS, DFS	0.31 (0.03~3.03)
Luo (2019) [14]	China	Tissue	qRT-PCR	100	50%	Median value	OS, DFS	1.91 (1.07~3.39)
Yu (2019) [15]	China	Tissue	qRT-PCR	392	15.31%	ROC	OS, CP	1.21 (0.97~1.50)
Guo (2020) [16]	China	Tissue	qRT-PCR	70	54.29%	Median value	OS, CP	1.24 (1.07~1.41)
Wang (2020) [17]	China	Blood	qRT-PCR	135	50.37%	Median value	OS, CP	2.73 (1.20~5.86)

### Meta-analysis results

### Analysis of the relationship between lncRNA NEAT1 expression status and the degree of differentiation of rectal cancer

**Figure 3:**
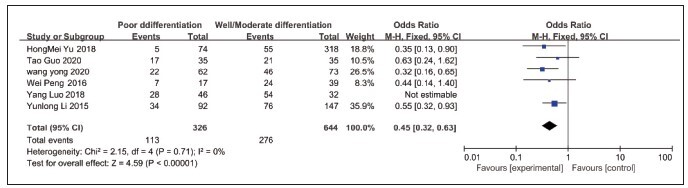
Forest plot of NEAT1 expression and the results of meta-analysis on the degree of differentiation of rectal cancer.

Six of the seven included papers had an association between lncRNA NEAT1 expression and rectal cancer differentiation, of which 276 patients were highly/moderately differentiated and 113 patients were poorly differentiated. There was no statistical heterogeneity (P=0.71, I2=0). Using a fixed effects model, the analysis showed [OR=0.45, 95%CI=0.32–0.63, P<0.01] that the difference was statistically significant, see Figure 3.

### Analysis of lncRNA NEAT1 expression profile in relation to tumour size

**Figure 4:**
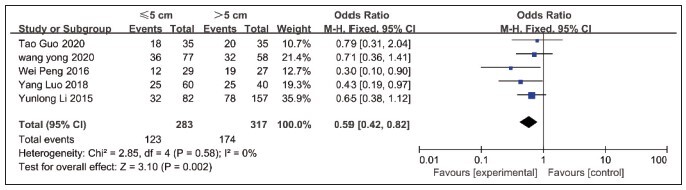
Forest plot of NEAT1 expression in relation to tumor size in meta-analysis.

The correlation between lncRNA NEAT1 expression and tumour size was reported in 5 papers. There was no statistical heterogeneity (*P*=0.58, I^2^=0). Using a fixed effects model, the analysis showed that the tumour diameter was greater in the high expression group, with a statistically significant difference [OR=0.59, 95% CI=0.42–0.82, *P*<0.01], see Figure 4.

### Analysis of the relationship between lncRNA NEAT1 expression and lymph node metastasis

**Figure 5:**
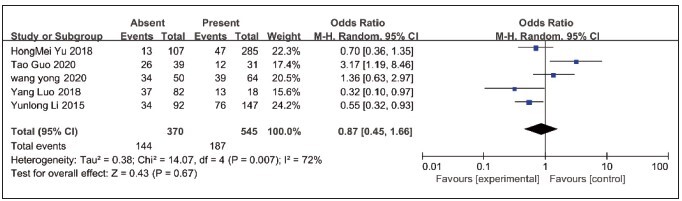
Meta-analysis of NEAT1 expression in relation to lymph node metastasis Forest plot.

The relationship between lncRNA NEAT1 expression and lymph node metastasis was reported in 5 papers with a total of 144 cases with lymph node metastasis and 187 cases without lymph node metastasis. There was statistical heterogeneity (*P*=0.007, I^2^=72). Using a random effects model, the results showed no statistically significant difference between groups [OR=0.87, 95% CI=0.45–1.66, *P*=0.67], see Figure 5.

### Results of the analysis of the correlation between lncRNA NEAT1 expression and gender

**Figure 6:**
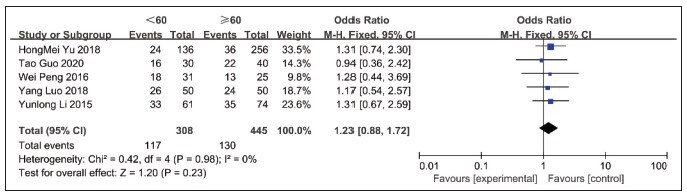
Forest plot of the results of the correlation between NEAT1 expression and gender meta-analysis.

The correlation between lncRNA NEAT1 expression and gender was reported in five papers, with 130 of the included NEAT1 high expression cases 60 years old and 117 cases <60 years old, with no statistical heterogeneity (*P*=0.98, I^2^=0). Using a fixed effects model, the analysis showed that the results showed no statistically significant differences between groups [OR=1.23, 95%CI=0.88-1.72, *P*=0.23], Figure 6.

### Analysis results of the relationship between lncRNA NEAT1 expression and OS

**Figure 7:**
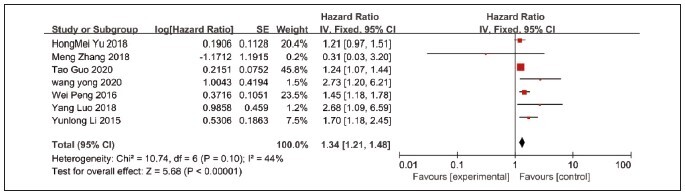
Forest plot of the results of meta-analysis on the relationship between NEAT1 expression and OS.

A total of 7 literatures reported the relationship between lncRNA NEAT1 expression and OS. Heterogeneity analysis showed that there was no high heterogeneity among the studies (I2=44%, P=0.01), so fixed effect model was used for quantitative analysis. The results of meta-analysis showed that the OS of patients with high NEAT1 expression in rectal tumors was lower than that of patients with low or no NEAT1 expression (HR=1.34, 95%CI=1.21–1.48), and the difference was statistically significant (P<0.001), Figure 7.

## Discussion

It has been found that lncRNAs can regulate gene expression at epigenetic, transcriptional and post-transcriptional levels and are closely associated with human diseases. lncRNAs are highly expressed in a variety of malignant tumours and are closely associated with patient prognosis, such as ALAT1, ANRIL, H19, CUDR, Zeb3 and other lncRNAs have been found to be highly expressed in lung cancer, breast cancer, colon cancer and other malignant tumors, playing the role of oncogenes, and are considered to be novel biomarkers for predicting the prognosis of human cancers [18]. There is now a wealth of data supporting the role of NEAT1 in cancer biology, including the observation that loss or attenuation of NEAT1 occurs in many human malignancies, including bone tumours, breast cancer, lung cancer, liver cancer, ovarian cancer, pancreatic cancer and many haematological disorders. NEAT1 is one of the most common oncogenes in lncRNAs, and its abnormal expression can contribute to the stem cell properties of tumour cells that facilitate the proliferation and metastasis of tumour cells, etc. [19]. Previous studies have analysed the relationship between NEAT1 and the prognosis of patients with gastrointestinal tumours, however, there are many limitations in this study. Specifically, firstly, the search was not comprehensive, articles after 2020 were not included, and new original studies on the relationship between NEAT1 and prognosis of patients with digestive tumours have emerged in recent years. This meta-analysis further improved the study design and search strategy to comprehensively include high-quality relevant original studies in order to improve the authenticity and reliability of the findings.

This meta-analysis included seven cohort studies including 1063 patients to investigate the role of NEAT1 in the prognosis of gastrointestinal malignancies. the results of the meta-analysis showed that it was combined in studies of the degree of differentiation with colorectal cancer (OR=0.45, *P*<0.01), in studies of the relationship with the presence or absence of lymph node metastasis (OR=0.59, *P*<0.01), and in studies of total survival studies (HR=1.34, *P*<0.001), patients in the high NEAT1 expression group had shorter OS with a combined effect size of [HR=1.34, 95%CI=1.21–1.48]. These findings suggest that NEAT1 could serve as a critical biomarker for predicting prognosis in rectal cancer patients. Additionally, the association of high NEAT1 expression with more aggressive cancer characteristics indicates a potential need for more tailored and possibly intensive treatment approaches for patients exhibiting high NEAT1 levels. This study underscores the significance of NEAT1 in rectal cancer prognosis and highlights the potential for developing NEAT1-targeted therapeutic strategies, thereby contributing to more effective patient management and treatment outcomes in rectal cancer care. However, this study still has some limitations. Firstly, the number of literature included in this meta-analysis was small; secondly, conducting the study solely in English limits its generalizability, as it excludes non-English research on NEAT1 in colorectal cancer. This exclusion risks bias and affects the comprehensiveness of conclusions, necessitating cautious interpretation. Future studies should include multilingual literature for a more global perspective finally, the search for this study was not comprehensive, as it excluded articles published after 2020. This limitation may impact the study’s findings, potentially omitting recent advancements and insights into NEAT1’s role in colorectal cancer, which could affect the overall conclusions and recommendations.

Based on the results of this study, it was demonstrated that lncRNA NEAT1 has a beneficial effect on the development of rectal malignancies, but the above findings are only limited to the meta-analysis. In addition, further studies are needed to determine whether the expression of lncRNA NEAT1 in various cancers is specific, and whether other factors are involved in the expression process and how the expression is affected. Unlike P53 and PTEN, which are more intensively researched genes, NEAT1 has been less studied in tumours at home and abroad, and the detailed role of NEAT1 in the development of tumours and its impact on the prognosis of tumour patients is only the tip of the iceberg, which needs to be studied continuously. The present analysis shows that NEAT1, as an oncogene, can inhibit the development of colorectal cancer, and when its expression is reduced, it can lead to the development of colorectal cancer and accelerate the infiltration and metastasis of colorectal cancer, which provides some help in the diagnosis and treatment of colorectal cancer. Therefore, further detection, analysis and study of the NEAT1 gene will provide a better understanding of the mechanism of colorectal cancer development, and will provide a more solid and reliable theoretical basis for the prevention and treatment of colorectal cancer.

The continuous research on NEAT1 is of paramount importance in enhancing our understanding of its role in colorectal cancer development. As a long noncoding RNA, NEAT1’s involvement in the molecular mechanisms of colorectal cancer remains a critical area of investigation. Further research into NEAT1 could reveal significant insights into tumor biology, potentially leading to breakthroughs in early diagnosis and more effective treatment strategies. By focusing on NEAT1, researchers can potentially identify novel biomarkers for colorectal cancer, aiding in the early detection and patient stratification. Additionally, understanding NEAT1’s specific functions could lead to the development of targeted therapies, offering more personalized and effective treatment options for patients. As such, NEAT1 research holds the promise of significantly improving the diagnosis, prognosis, and treatment of colorectal cancer, ultimately contributing to better patient outcomes and advancements in cancer therapy.

## Dodatak

### Authors’ contributions

Guoxi Xu and Qiyi Lin designed the study and performed the experiments, Jianpeng Pan, Huaishuai Wang and Yinlin Li collected the data, Yixiang Zhuang, Jianpeng Chen, Gaofeng Lin and Weibo Liu analyzed the data, Guoxi Xu prepared the manuscript. All authors read and approved the final manuscript.

### Acknowledgments

This work was supported by the Science and Technology Project of Jinjiang City Hospital (Shanghai Sixth People’s Hospital Fujian Hospital) (Project number: 2022JC01), Project name: Study on the expression of ASPN in gastric cancer and its mechanism of action with macrophage polarization.

### Conflict of interest statement

All the authors declare that they have no conflict of interest in this work.

## References

[1] Bray F, Ferlay J, Soerjomataram I, Siegel R L, Torre L A, Jemal A (2018). Global cancer statistics 2018: GLOBOCAN estimates of incidence and mortality worldwide for 36 cancers in 185 countries. Ca-Cancer J Clin.

[2] Wu C, Li M, Meng H, Liu Y, Niu W, Zhou Y, et al (2019). Analysis of status and countermeasures of cancer incidence and mortality in China. Sci China Life Sci.

[3] 3. *** (2020). Erratum: Global cancer statistics 2018: GLOBOCAN estimates of incidence and mortality worldwide for 36 cancers in 185 countries. Ca-Cancer J Clin.

[4] Jocić M, Arsenijević N, Gajović N, Jurišević M, Jovanović I, Jovanović M, Zdravković N, Marić V, Jovanović M (2022). Anemia of inflammation in patients with colorectal cancer: Correlation with interleukin-1, interleukin-33 and galectin-1. J Med Biochem.

[5] Ji P, Diederichs S, Wang W, Boing S, Metzger R, Schneider P M, et al (2003). MALAT-1, a novel noncoding RNA, and thymosin beta4 predict metastasis and survival in early-stage non-small cell lung cancer. Oncogene.

[6] Hutchinson J N, Ensminger A W, Clemson C M, Lynch C R, Lawrence J B, Chess A (2007). A screen for nuclear transcripts identifies two linked noncoding RNAs associated with SC35 splicing domains. Bmc Genomics.

[7] Klec C, Prinz F, Pichler M (2019). Involvement of the long noncoding RNA NEAT1 in carcinogenesis. Mol Oncol.

[8] Lo P K, Wolfson B, Zhou Q (2016). Cellular, physiological and pathological aspects of the long non-coding RNA NEAT1. Front Biol (Beijing).

[9] Yu X, Li Z, Zheng H, Chan M T, Wu W K (2017). NEAT1: A novel cancer-related long non-coding RNA. Cell Proliferat.

[10] Li Y, Li Y, Chen W, He F, Tan Z, Zheng J, et al (2015). NEAT expression is associated with tumor recurrence and unfavorable prognosis in colorectal cancer. Oncotarget.

[11] Higgins J P, Altman D G, Gotzsche P C, Juni P, Moher D, Oxman A D, et al (2011). The Cochrane Collaboration's tool for assessing risk of bias in randomised trials. Bmj-Brit Med J.

[12] Peng W, Wang Z, Fan H (2017). LncRNA NEAT1 Impacts Cell Proliferation and Apoptosis of Colorectal Cancer via Regulation of Akt Signaling. Pathol Oncol Res.

[13] Zhang M, Weng W, Zhang Q, Wu Y, Ni S, Tan C, et al (2018). The lncRNA NEAT1 activates Wnt/beta-catenin signaling and promotes colorectal cancer progression via interacting with DDX5. J Hematol Oncol.

[14] Luo Y, Chen J J, Lv Q, Qin J, Huang Y Z, Yu M H, et al (2019). Long non-coding RNA NEAT1 promotes colorectal cancer progression by competitively binding miR-34a with SIRT1 and enhancing the Wnt/beta-catenin signaling pathway. Cancer Lett.

[15] Yu H M, Wang C, Yuan Z, Chen G L, Ye T, Yang B W (2019). LncRNA NEAT1 promotes the tumorigenesis of colorectal cancer by sponging miR-193a-3p. Cell Proliferat.

[16] Guo T, Liu D F, Peng S H, Xu A M (2020). ALKBH5 promotes colon cancer progression by decreasing methylation of the lncRNA NEAT1. Am J Transl Res.

[17] Wang Y, Zhang D, Zhang C, Sun Y (2020). The Diagnostic and Prognostic Value of Serum lncRNA NEAT1 in Colorectal Cancer. Cancer Manag Res.

[18] Silva J, Da C M P (2022). Non-Coding RNAs in the Therapeutic Landscape of Pathological Cardiac Hypertrophy. Cells-Basel.

[19] Feng Y, Gao L, Cui G, Cao Y (2020). LncRNA NEAT1 facilitates pancreatic cancer growth and metastasis through stabilizing ELF3 mRNA. Am J Cancer Res.

